# SSRI and Motor Recovery in Stroke: Reestablishment of Inhibitory Neural Network Tonus

**DOI:** 10.3389/fnins.2017.00637

**Published:** 2017-11-16

**Authors:** Camila B. Pinto, Faddi G. Saleh Velez, Fernanda Lopes, Polyana V. de Toledo Piza, Laura Dipietro, Qing M. Wang, Nicole L. Mazwi, Erica C. Camargo, Randie Black-Schaffer, Felipe Fregni

**Affiliations:** ^1^Laboratory of Neuromodulation and Center for Clinical Research Learning, Department of Physical Medicine and Rehabilitation, Harvard Medical School, Spaulding Rehabilitation Hospital, Harvard University, Boston, MA, United States; ^2^Department of Neuroscience and Behavior, Psychology Institute, University of São Paulo, São Paulo, Brazil; ^3^Department of Severe Patients, Hospital Israelita Albert Einstein, São Paulo, Brazil; ^4^Highland Instruments, Cambridge, MA, United States; ^5^Stroke Biological Recovery Laboratory, Department of Physical Medicine and Rehabilitation, Harvard Medical School, Spaulding Rehabilitation Hospital, Harvard University, Boston, MA, United States; ^6^Department of Physical Medicine and Rehabilitation, Harvard Medical School, Spaulding Rehabilitation Hospital, Harvard University, Boston, MA, United States; ^7^Department of Neurology, Harvard Medical School, Massachusetts General Hospital, Harvard University, Boston, MA, United States

**Keywords:** stroke, motor rehabilitation, SSRIs, cortical excitability, inhibitory tonus, neuroplasticity

## Abstract

Selective serotonin reuptake inhibitors (SSRIs) are currently widely used in the field of the neuromodulation not only because of their anti-depressive effects but also due to their ability to promote plasticity and enhance motor recovery in patients with stroke. Recent studies showed that fluoxetine promotes motor recovery after stroke through its effects on the serotonergic system enhancing motor outputs and facilitating long term potentiation, key factors in motor neural plasticity. However, little is known in regards of the exact mechanisms underlying these effects and several aspects of it remain poorly understood. In this manuscript, we discuss evidence supporting the hypothesis that SSRIs, and in particular fluoxetine, modulate inhibitory pathways, and that this modulation enhances reorganization and reestablishment of excitatory-inhibitory control; these effects play a key role in learning induced plasticity in neural circuits involved in the promotion of motor recovery after stroke. This discussion aims to provide important insights and rationale for the development of novel strategies for stroke motor rehabilitation.

## Introduction

Stroke is the second cause of death worldwide and the leading cause of death in upper-middle income countries; about 6.7 million people died from stroke in 2013 in the US (Mozaffarian et al., 2016). Among stroke survivors, recovery of motor function is frequently incomplete, with the majority of stroke patients unable to perform professional duties or activities of daily living 6 months after the event (Hummel and Cohen, 2005, 2006).

Current conventional therapies rely on behavioral treatments such as physiotherapy and occupational therapy (Winstein et al., 2016); however, these treatments only induce limited plastic and cortical reorganization changes (Veerbeek et al., 2014). Thus, considerable research efforts have been devoted to developing methods for enhancing neuroplasticity in stroke and increasing the efficacy of rehabilitation techniques.

To this end, the use of pharmacological agents such as selective serotonin reuptake inhibitors (SSRIs), in particular fluoxetine, is increasingly being explored by several research groups. It has been shown that SSRIs can modulate neural excitability, promote plastic changes and improve motor rehabilitation after stroke (Chollet et al., 2011; Siepmann et al., 2015). Chollet et al. reported positive results of the Flame (fluoxetine for motor recovery after acute ischemic stroke) trial. They hypothesized that fluoxetine enhances motor recovery after stroke through a coupling of its neuroprotective effect with the serotonergic system capability to enhance motor outputs and facilitate long term potentiation (LTP) (Chollet et al., 2011). The encouraging results of the Flame trial were further supported by the positive results of a Cochrane review of SSRIs for stroke recovery including 52 trials and 4,059 patients (Mead et al., 2013).

The similarity between the processes of stroke recovery and learning has also recently gained increasing attention, and it is being explored by several research groups with the goal of improving the therapeutic efficacy of technologies for stroke rehabilitation (Hogan et al., 2006; Krakauer, 2006). Neural repair and recovery after stroke involve mechanisms of neuronal excitability modulation that are very similar to those involved in memory and learning processes (Krakauer, 2006).

Although their exact function during these processes is still unclear (Clarkson et al., 2010), the important role of inhibitory networks has begun to be acknowledged. These networks are thought to play a key role in both early and late stages of learning-induced plastic circuits. In fact, a strong neural system to promote learning is a system that can rapidly shift between inhibition and excitation (Trevelyan, 2016).

While the exact mechanisms of action of SSRIs on the neural system after stroke are far from being understood, this review aims to discuss the potential role of SSRIs on inhibitory neural circuits as a possible mechanism underlying motor rehabilitation after stroke through cortical reorganization and enhancement of motor learning. We critically analyze evidence regarding: (i) The relationship between inhibitory neural activity and motor learning; (ii) Disruption of inhibitory activity after stroke; (iii) Evidence of SSRIs-induced enhancement of inhibitory tone; and (iv) Evidence of SSRIs-induced enhancement of motor learning through an increase of inhibitory tone in stroke.

### Relationship between inhibitory neural activity and motor learning

Motor learning leads to alteration in the cortical motor mapping, promoting changes in the motor and somatosensory representations. Motor learning involves neuronal excitability and inhibitory modulatory mechanisms that are very similar to the mechanisms involved in memory and non-motor learning processes (Krakauer, 2006).

Learning a new motor skill shares similar physiological traits with motor recovery from stroke. The similarities between these processes provide us with a model that can be applied to certain processes of functional recovery after stroke (Krakauer, 2006; Rossi et al., 2009; Krakauer and Mazzoni, 2011; Kitago and Krakauer, 2013; Costanzo, 2017). For instance, the modifications of motor behavior after exposure to stimuli or experiences (learning) are similar to those occurring during physical therapy; however, in the latter case the modifications underlie a process of re-learning of behaviors lost due to the structural alteration caused by the cerebrovascular accident (Krakauer, 2006; Krakauer and Mazzoni, 2011).

Additionally, memory plays an important role in the consolidation of newly learned behaviors, therefore the enhancement of mechanisms underlying both learning and memory is crucial for therapies aimed at promoting stroke motor recovery (Krakauer, 2006).

The neurophysiological mechanism underlying learning and memory is synaptic plasticity. Enhanced and more effective synaptic functions largely depend on facilitating the likelihood of neuronal inputs to happen; repeated stimuli generate a pattern of repeated activation on a determined pathway leading to an enhanced responsiveness of the post synaptic neuron (potentiation) (Feldman, 2009; Costanzo, 2017). On a cellular level these processes depend on changes in network excitability as regulated by long-term potentiation (LTP) and long-term depression (LTD) (Hagemann et al., 1998). LTP and LTD can induce changes in synaptic strengthening, thereby shaping learning and memory processing (Bliss and Collingridge, 1993; Lynch, 2004).

**Figure 1:**
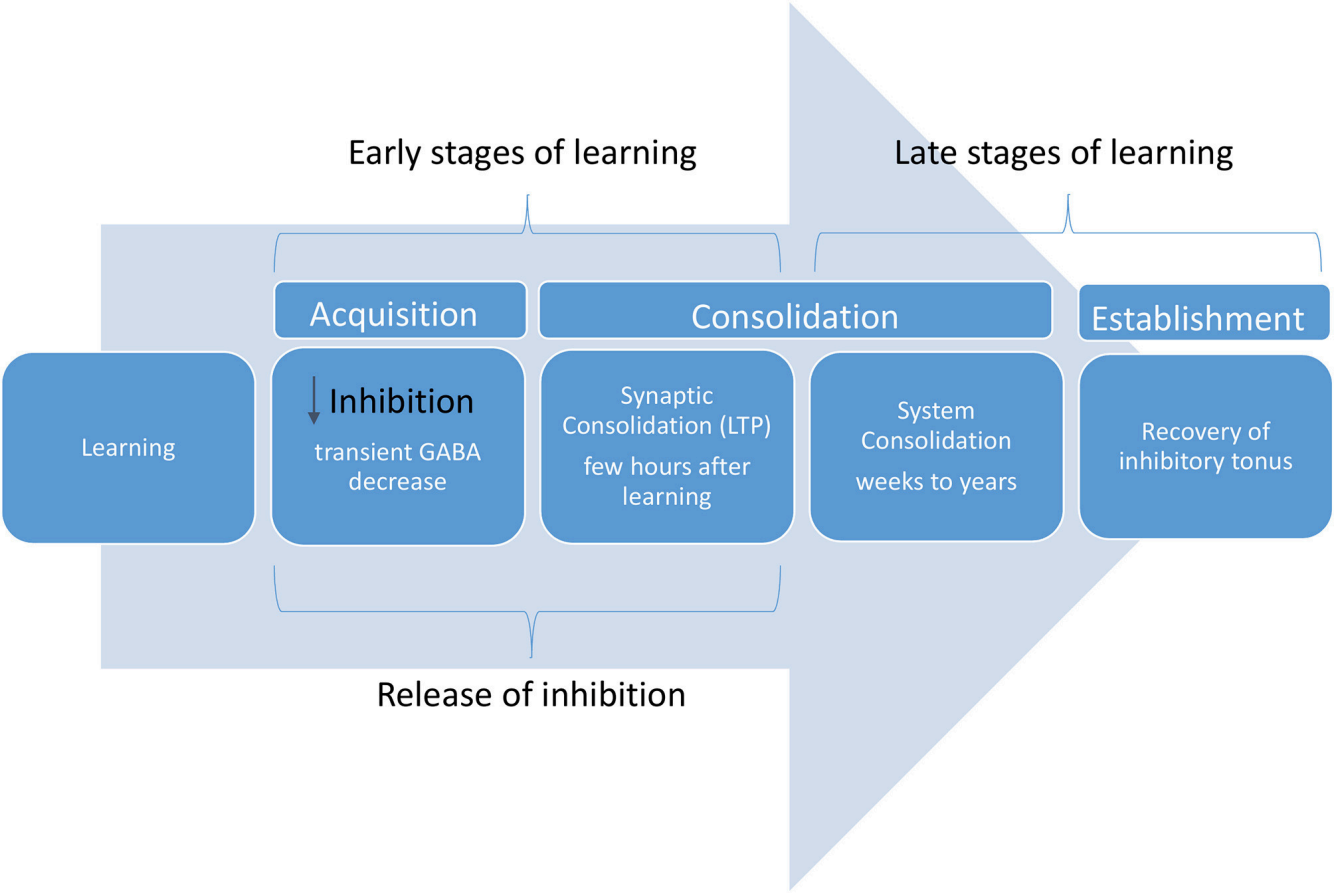
Neurobiology of learning phases.

Although little is known about the spatiotemporal aspects of motor learning (Berger et al., 2017), the modulation of inhibitory activity is an important mechanism underlying motor cortex plasticity. In fact, inhibitory activity can change during early and late stages of the learning process (See Figure 1; Engel et al., 2001; Hensch and Stryker, 2004; Stagg et al., 2011).

The early stage of learning is characterized by reduction in inhibition, facilitating LTP process in the motor cortex (Nudo et al., 1996; Floyer-Lea et al., 2006). Local changes in inhibitory circuits (GABA concentrations) can unmask existent neural pathways within the cortex leading to rapid changes in the sensory and motor representations (Castro-Alamancos et al., 1995; Castro-Alamancos and Connors et al., 1996). It has been shown that during early learning a transient GABA decrease in the motor cortex correlates with the degree of motor learning (Floyer-Lea et al., 2006; Stagg, 2013). Despite the transient impact of lower inhibitory activity in acute phases of learning, this correlation may be inverted in late phases of learning consolidation.

In later stages of motor learning, a partial recovery in inhibitory tonus (restoration of the inhibitory tonus) can be associated with longer-term consolidation of learning (Shadmehr and Holcomb, 1997; Floyer-Lea et al., 2006).

An interesting study using TMS, tDCS and neuroimaging showed that the degree of learning is associated with a decrease in GABA activity induced by tDCS (Johnstone et al., 2016), thus supporting the notion that during early learning stages a decreased and transient inhibitory tone is desirable. However, subsequent inhibition seems necessary for consolidation of learning (Brosh and Barkai, 2009).

Taken all together, these studies suggest that understanding the balance between excitation and inhibition activity during motor learning is key to better understanding the process of motor learning. Assuming that stroke recovery and motor learning are somewhat similar processes (Hogan et al., 2006; Krakauer, 2006), this knowledge may help understand how to improve rehabilitation in stroke patients.

### Disruption of inhibitory activity after stroke

Following stroke, several structural and biological changes take place and the interactions between excitatory and inhibitory activity in learning-related circuits are crucial to drive motor cortex plasticity and recovery (Jones, 2017). In the very acute phase of stroke (hours after the event), inhibition of activity of cortical areas surrounding the injured site is a neuroprotective mechanism since increased excitability can result in glutamatergic toxicity and increase apoptosis (Schiene et al., 1996; Floyer-Lea et al., 2006).

After this initial phase, and similarly to early stages of learning, the excitation/inhibition ratio increases (Schiene et al., 1996). Two main neurophysiological changes have been associated with brain remodeling and recovery after stroke: (i) an initial increase in excitability in cortical regions and perilesional areas not affected by the stroke, and (ii) increased activity/excitability in the contralesional hemisphere. In this context, several studies showed that in stroke patients the inhibitory function is globally reduced in both hemispheres suggesting that the modulation of inhibitory circuits might result from a strategy that aims at compensating for motor impairment (Talelli et al., 2006; Di Lazzaro et al., 2012; Takeuchi and Izumi, 2012). Even though this enhanced excitability pattern is related to the enhanced plasticity and functional reorganization, both changes are required to be time dependent.

In the peri-lesional and distant cortical regions the down-regulation of the inhibitory tonus is important for altering cortical maps and promoting neuronal connectivity (*re-hardwiring*) (Schiene et al., 1996). Moreover, manipulation of the inhibitory tone promotes axonal and dendritic growth and guidance, cytoskeletal organization and the expression of genes related with adult brain remodeling (Urban et al., 2012; Nudo, 2013).

Widespread areas of enhanced cortical excitability appear after stroke, and are important for some of the compensatory re-organization (Collins, 2016). For example, Frost et al. (2003) showed that in adult squirrel monkeys an increased hand representation in ventral pre-motor areas (PMv) is observed after an experimental ischemic lesion of 50% of the M1 hand area. Additionally, the amount of reorganization was correlated with the size of the hand area lesion, suggesting that bigger lesions induce greater compensatory reorganization (Frost et al., 2003). Although the development of compensatory reorganization results in initial gain of function, it may inhibit further gain of function in later phases of stroke rehabilitation (Meintzschel and Ziemann, 2006; Carmichael, 2012).

In this regard, increased disinhibition of the widespread cortical areas increases the risk of competitive interaction between the areas (pre motor cortex and M1), thereby resulting in incomplete motor recovery (Takeuchi et al., 2007). On the other hand, the localized and transient disinhibition of the affected M1 in stroke patients may promote the motor recovery of normal patterns by facilitating M1 plasticity (Takeuchi and Izumi, 2012).

One example is the surrounding inhibitory theory that highlights the importance of inhibitory networks during the initiation of active voluntary tasks. During movement preparation, there is a local decrease of inhibition (decrease in GABAa) and a concomitant increase of inhibition in the brain neighboring areas; these processes avoid competition between local and neighboring areas and allow specific activation of the motor area in charge of generating the desired motor behavior (Pfurtscheller et al., 2005; Klimesch, 2012; Pfurtscheller and Lopes da Silva, 2017). In stroke patients, the local decrease of inhibitory activity prior to movement is reduced and there is a widespread disinhibition in the neighboring areas of the lesion (Reynolds and Ashby, 1999; Zaaroor et al., 2003; Hummel et al., 2009).

Therefore, stronger functional recovery is associated with a time-dependent increase of inhibition. In fact, an important aspect that has not been fully explored in human studies is the modulation of the inhibitory states; varying from disinhibition to inhibition in cortico-subcortical circuits according to the phase of learning. A shift in one direction only (i.e., disinhibitory state) has a low learning efficiency. In fact, that shift may be an index of cortical disorganization.

### A network example of disrupted excitation-inhibition balance: transcallosal imbalance and motor recovery in stroke

An example of imbalance in the excitation-inhibition ratio can be seen in the transcallosal interaction between motor cortices in stroke (Julkunen et al., 2016). Functional imaging and non-invasive brain stimulation have indicated that ipsilateral motor projections are enhanced early after stroke and that a pattern of hyper-excitation is commonly observed in the unaffected hemisphere (Turton et al., 1996; Netz et al., 1997; Werhahn et al., 2003; Murase et al., 2004; Oh et al., 2010). Although the underlying mechanism remains unclear, it is believed that latent ipsilateral motor projections are activated by disruption of the contralateral corticospinal projections in stroke patients (Feydy et al., 2002; Johansen-Berg et al., 2002). This results in the reconfiguration of motor networks through the establishment of alternative inputs for the cortico-spinal tracts. Then, parallel motor circuits are activated and begin to transfer the impaired functions to unaffected areas of the brain (Feydy et al., 2002; Lindenberg et al., 2010).

In later stages of stroke recovery, better motor recovery and outcome of functional rehabilitation are associated with a shift of activation back to the affected hemisphere by the reestablishment of the inhibitory tonus in the unaffected hemisphere as well as with recovery of connectivity and cessation of imbalance between the two hemispheres (Jaillard et al., 2005; Carter et al., 2010; Park et al., 2011).

A persistent increased activity of the unaffected hemisphere can result in alterations of transcallosal structure and function and it is associated with upper limb motor impairment and greater levels of arm motor dysfunction (Sato et al., 2015) in chronic stroke patients. In this context, several studies showed the importance of inhibition after stroke and the association between increased inhibition of the unaffected hemisphere with better functional outcomes (Conforto et al., 2008; Swayne et al., 2008). Additionally, patients with severe upper limb impairment display higher disinhibition of the unaffected hemisphere compared to patients with mild-moderate impairment (Liepert et al., 2000).

**Figure 2:**
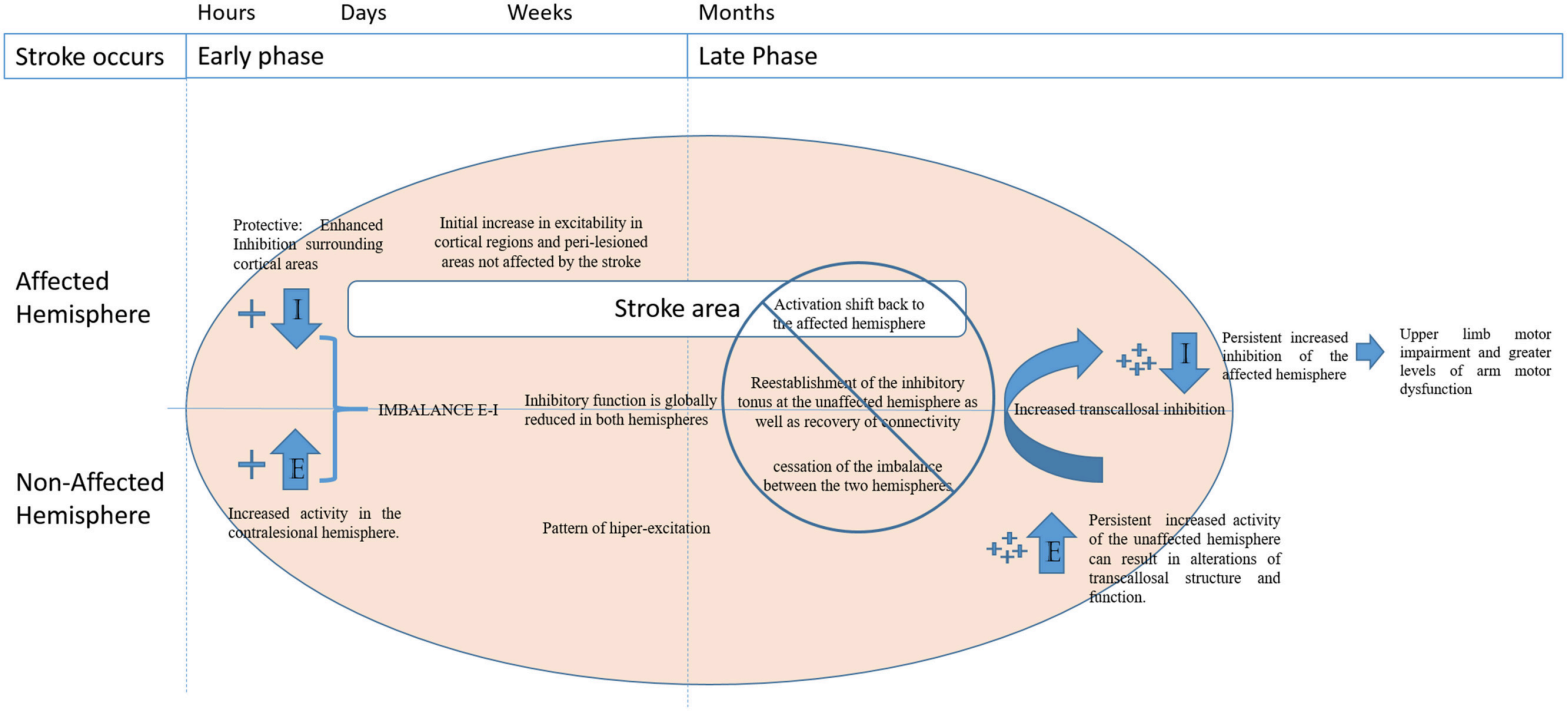
Disruption of balance between excitation (E) and inhibition (I) of the affected and non-lesioned hemispheres throughout the time post stroke.

Therefore, it is hypothesized that the balance between excitatory and inhibitory inputs after stroke is crucial for long term stroke recovery and motor re-learning. These time dependent changes in balance between excitatory and inhibitory networks are also seen in healthy subjects (Guerra et al., 2016). However, after a stroke this process can be altered and jeopardize motor recovery. Consequently, longitudinal changes in the regulation of inhibitory functions seem to play a major role in this balance, since the lack of inhibition in later phases of stroke recovery is associated with worse motor function outcomes (see Figure 2; Crichton et al., 2016).

### Can SSRIs enhance inhibitory tone?

SSRIs are the most common treatment for major depressive disorder (MDD). Clinical and preclinical data of MDD patients indicate imbalanced activity in the right dorsolateral prefrontal cortex (DLPC) compared to the left cortex/counterpart (Grimm et al., 2008). It has been shown that impaired inhibition of the right hemisphere, as indexed by TMS measures such as intracortical inhibition (ICI), intracortical facilitation (ICF) and cortical silent period, is correlated with treatment responsiveness and recovery after depression (Mantovani et al., 2012). Several authors have also demonstrated that adults with MDD present deficits in GABAergic mediated transmission related with the severity of MDD (Robinson et al., 2003; Ye et al., 2008; Croarkin et al., 2014). Moreover, it has been shown that acute (intravenous infusion) (Bhagwagar, 2004) or chronic (2 months) (Sanacora et al., 2002) administration of SSRIs can increase cortical levels of GABA in depressed adults, leading to the normalization of GABA levels and possibly contributing to an antidepressant mechanism.

Besides that, in mouse models of social isolation, fluoxetine treatment can induce stress recovery independently of its serotonergic pathways. In these models, fluoxetine acts by increasing the neurosteroids levels in the brain allowing a normal allosteric increase of GABA-A synapses and therefore restoring inhibition (Matsumoto et al., 2007).

Several neurological conditions seem to depend on a balance between excitatory and inhibitory activity in the brain, and the inhibitory control of excitation may have a key role in this balance. Dysfunction in GABA-controlled inhibition is pathophysiologically correlated with several diseases and restoration of functional inhibition leads to better treatment outcomes. In particular, fluoxetine was shown to have positive effects on motor rehabilitation after stroke and preclinical data demonstrated that fluoxetine can directly up regulate GABA, increasing inhibitory activity (Tunnicliff et al., 1999; Robinson et al., 2003). In fact it is possible that one of the mechanisms of SSRIs in improving depression is through a reestablishment of normal learning patterns of prefrontal circuits.

### SSRIs and changes in inhibitory activity in stroke

Inhibitory circuits play an essential role in post-stroke motor learning. SSRIs seem to exert an effect on excitation/inhibition pathways, leading to enhancement of motor function after a stroke. Although this mechanism is still underexplored in stroke, other disease models are clearer; some insights from these conditions can be extrapolated to better understand the potential role of this class of drugs in stroke patients.

Recent studies have shown the effects of drugs that are capable of modulating excitatory and inhibitory pathways and this may be how they facilitate functional recovery after brain lesions. Some of these pharmacological candidates are SSRIs (Pariente et al., 2001; Chollet et al., 2011), dopamine agonist (Scheidtmann et al., 2001), amphetamines (Bütefisch et al., 2002; Sawaki et al., 2002b) and cholinergic substances (ACR) (Sawaki et al., 2002a; Meintzschel and Ziemann, 2006).

Among these drugs, SSRIs have been shown to be able to produce different effects (inhibitory or excitatory) on motor cortex plasticity (Robol et al., 2004). The neuroplastic changes triggered by SSRIs can be responsible for the compensation of some of the neurophysiologic damage caused by the stroke. Several early studies suggested that serotonergic agents such as fluoxetine and citalopram have a positive effect on motor function in hemiplegic patients after stroke (Dam et al., 1996; Pariente et al., 2001; Zittel et al., 2008). The Flame trial, a large randomized placebo-controlled clinical trial, showed that the administration of fluoxetine during the acute stroke phase has a moderate effect size, resulting in improvement of a clinical motor function scale for upper and lower limbs outcome (Fugl-Meyer) (Chollet et al., 2011).

To date, the mechanism by which fluoxetine enhances rehabilitation after stroke remain unclear; however, some insights from animal-based models suggested that SSRIs may enhance overall brain excitability. Additionally, it has been shown that SSRIs can promote neuronal sprouting (Kokoeva et al., 2005; Hourai and Miyata, 2013; Ohira et al., 2013b) and cortical reorganization (26), restore blood flow (Rosenstein et al., 1998; Sun et al., 2003), and enhance neuroprotective mechanism (Lim et al., 2009); thereby improving neuronal survival and protecting cerebral tissue from hypoxia since it regulates the expression of hypoxia-inducible factor-1α (HIF-1α) and of heme oxygense-1 (HO-1) (Shin et al., 2009; Siepmann et al., 2015). Therefore, one hypothesis for the motor rehabilitation induced by fluoxetine is that the blockage of serotonin (5-HT) reuptake due to SSRI administration increases the availability of this neurotransmitter in the synaptic cleft thus enhancing signal transmission (Ganzer et al., 2013; Rief et al., 2016; Stahl, 2017) and consequently, increasing the excitatory input of glutamate and activating the NMDA receptors leading to a cascade of intracellular events. These events might generate synaptic modifications that culminate in a LTP leading synapse reprogramming and strengthening (Stahl, 2017).

Nevertheless, we also believe that this acute effect on enhancement of excitatory activity is followed by an increase in inhibitory activity. Studies exploring the effects exerted by SSRIs over motor cortex plasticity showed that SSRIs can also enhance inhibitory activity (Etherton et al., 2016). Studies have shown that serotonergic drugs induce changes in inhibitory activity. Indeed inhibition in the motor cortex was observed in healthy subjects 2.5 h after citalopram intake. A single dose of citalopram produced a transient enhanced GABAergic interneuronal control over corticospinal neurons, resulting in increases in the ICI, motor threshold and cortico-silent period (CSP) as well as mild decrease in the ICF (Robol et al., 2004). Therefore, citalopram was able to acutely decrease motor cortex excitability in healthy subjects.

In addition, pre-clinical data suggest that citalopram, fluoxetine and sertraline have anticonvulsive effects in the hippocampus inhibiting seizure like events. The effects of these SSRIs were likely due to regulation of pyramidal cell's excitability leading to vigorous inhibition of repetitive firing (Igelström and Heyward, 2012). However, in clinical studies an increase in seizures after administration of SSRIs was also described (Hill et al., 2015).

Recently, authors have been exploring the role of long term administration of fluoxetine treatment administration (Chen et al., 2011) inducing structural plasticity by promoting neuron “dematuration” (Kobayashi et al., 2010; Ohira et al., 2013a,b). This mechanism consists in a reverse effect on the normal maturate state of cells that can be induced by changes in GABAergic transmission; therefore, culminating in a juvenile-like state of fast-spiking inhibitory interneurons. In several studies, neuronal “dematuration” induced by fluoxetine was identified in different brain regions such as adult dentate granule cells, adult amygdala cells (Karpova et al., 2011), hippocampus, medial pre-frontal cortex (mPFC) (Guirado et al., 2014) and a subset of GABAergic interneurons, generated from Layer 1 inhibitory neuron progenitor cells (L1-INP cells) (Ohira et al., 2013a).

In this context, it is important to acknowledge that the same antidepressant can exert different regulatory effects in different brain regions and these effects vary depending on dose, regimen of use (single dose or prolonged use) and time of administration (in acute or chronic diseases phase). For example, a single dose of paroxetine can improve motor performance and induce hyper activation of the primary sensorimotor cortex (S1M1) (Loubinoux et al., 2005). However, prolonged paroxetine treatment (30 days 20 mg per day) induced hypo activation of S1M1 cells, but still resulted in improvement in finger tapping motor task (Gerdelat-Mas et al., 2005). These results indicate that SSRIs may have an acute effect on excitatory circuits that is led by enhancement of inhibitory activity.

Even though the exact mechanism by which this effect occurs is unknown, prior studies showed that some SSRIs can potentiate GABA-mediated inhibitory neurotransmission. Among SSRIs, fluoxetine's mechanism of action includes its 5-HT2C antagonism. Furthermore, 5-HT2C receptors are expressed on GABAergic neurons (Serrats et al., 2005; Invernizzi et al., 2007).

Furthermore, SSRIs can activate other receptors such as the 5-HT2B receptor that have been identified in multiple brain cells such as neurons, astrocytes, and Purkinje cells (Chen et al., 1995; Peng et al., 2014). However, animal studies have shown that its expression can be twice as much in astrocytes than in neurons (Lovatt et al., 2007; Dong et al., 2015). Astrocytes have a main role in the neuronal metabolic process in the brain and recent evidence shows that fluoxetine can alter this metabolic process through 5-HT2B receptor activity (Peng et al., 2014; Hertz et al., 2015a).

The activation of 5-HT2B leads to a cascade of effects that have been associated with the enhancement of learning and memory. Among those processes, animal studies showed that its activation can lead to raise of intracellular calcium levels in astrocytes and consequent increase of glycogenolysis and glycolysis, causing subsequent increase of lactate release that can either act as extra metabolic fuel for neurons or act in further signaling pathways (Li et al., 2011; Hertz et al., 2015a; Steinman et al., 2016). Other processes such as potassium homeostasis are also enhanced by this activation (Du et al., 2014). In this regard, it is known that lactate has a neuroprotective effects during brain ischemia and that glycogen metabolism have many implications for the brain activities. In addition, it is suggested that the 5-HT2B receptor plays an important role in neurogenesis driven by SSRI treatments since studies in animals lacking this receptor revealed a deficient response to the enhanced effects of fluoxetine in neurogenesis (Diaz et al., 2012, 2016).

Moreover, the effects exert by fluoxetine differ depending on its dose as different responses are seen in glycogen concentration in astrocytes with different doses of fluoxetine. Whereas lower concentration of fluoxetine causes an increase of glycogen in astrocytes the opposite effects are seen with an increased concentration of the drug. It is believe that this dual effect can be associated with aspects of either the therapeutic effect or adverse event of the medication (Bai et al., 2017). The effects of fluoxetine in the 5-HT2B receptor need to be further explored in human studies since its role in plasticity can be used to better explain the mechanism underling this drug effects in neuroplasticity and motor rehabilitation after stroke.

In this context, the astrocytic metabolism could have a role in regulation excitatory and inhibitory mechanism after stroke. The activation of GABA-A receptors in astrocytes can stimulate a similar mechanism enhancing regulation of calcium concentrations and further enhancement of glycogenolysis, a process that has already been associated with learning and memory tasks; therefore relevant in the setting of motor recovery (Hertz et al., 2015b). Finally, the effects of fluoxetine in the 5-HT2B receptor need to be further explored in human studies since its role in plasticity can be used to better explain the mechanism underlying this drug effects in neuroplasticity and motor rehabilitation after stroke.

### SSRIs and safety considerations

Most studies to date have reported little or no side effects in relation of SSRI use in the stroke population; however, known potential adverse effects on mood disorders should be discussed. Due to their selective action, SSRIs present a more tolerable profile of adverse actions and there are differences between the main side effects of the different selective serotonin reuptake inhibitors. Overall, the most frequently reported adverse events are nausea, vomiting, abdominal pain, diarrhea, agitation, anxiety, insomnia, cycling for mania, headache, dizziness, fatigue, tremors, extrapyramidal effects, xerostomia, perspiration, loss or weight gain, sexual dysfunction, and dermatological reactions.

Moreover, SSRIs effects on patient with pre-existent cardiac disease or acute coronary syndrome are controversial as they may improve cardiac disease prognosis by inhibiting platelet aggregation but could potentially worsen prognosis by increasing risk of bleeding or of arrhythmias (Rieckmann and Kronish, 2013).

Another important aspect associated with increased serotonergic activity in the central nervous system is the Serotonergic syndrome, a potentially fatal condition (Peng et al., 2014). The most common symptoms include: hyperthermia, agitation, ocular clone, tremor, akathisia, deep tendon hyperreflexia, inducible or spontaneous clonus, muscular rigidity, dilated pupils, dry mucous membranes, increased intestinal sounds, spotted skin and diaphoresis (Boyer and Shannon, 2005). Although, serotonergic syndrome is commonly the result of the combination of SSRIs with drugs or dietary supplements that have a similar effect of increasing serotonin, it may occur after the initiation of a higher doses of single serotonergic drugs or increase of the dose of a serotonergic drug in particularly sensitive individuals. Selective serotonin reuptake inhibitors, such as fluoxetine, are perhaps the most implicitly associated drug group associated with serotonergic syndrome. They may contribute to the development of serotonin syndrome up to several weeks after drug discontinuation (Birmes et al., 2003).

Even though uncommon, the FLAME trial reported that among the 118 participants two in the active fluoxetine group have severe adverse events (hyponatremia and partial seizure). The most frequent adverse events in the active group were transient digestive disorders. To avoid serotonin syndrome, treatment was stopped in patients in the active group who developed depression and required the use of another antidepressant during the study (Chollet et al., 2011). Therefore, if this hypothesis that SSRIs improve motor function by modulating the excitatory-inhibitory balance is confirmed, other interventions that are safer may be best better first alternatives to SSRIs in this context.

## Summary

The mechanisms underlying the enhancement of motor learning via SSRIs are still unclear, and the exact effects of fluoxetine in motor recovery after stroke are yet to be proven. We discussed how this class of drugs might help reestablish the excitation-inhibitory balance by initially enhancing excitation that is followed by a compensatory inhibition. Different techniques such as functional magnetic resonance, magnetic resonance spectroscopy, transcranial magnetic stimulation, transcranial direct current stimulation and EEG have been used to show the role of neurochemical and neurophysiological markers as well as to quantify modifications of inhibitory and excitatory responses to several interventions. However, further work needs to be done to develop better methods to assess specific alterations in inhibitory pathways.

Ultimately SSRIs can have a broader action on promoting reorganization of neural activity following brain lesion. With the current evidence it is not possible to conclude whether the increased neural inhibition that follows use of SSRIs is indeed the result of its usage. However further studies can explore further this mechanism as to understand better how to use SSRIs and the potential limitations of this class of drugs that may also be detrimental in some cases.

## Author contributions

CP: conception and design of the manuscript, acquisition of data, analysis and interpretation of data reviewed, drafting revising and important intellectual contribution, critical revision and final approval of the version; FS: conception and design of the manuscript, interpretation of data reviewed, drafting revising and important intellectual contribution, and critical revision; FL and PdTP: acquisition of data, analysis, and final review; LdP, NM, RB-S, QW, and EC: critical revision, review, and final approval of the version; FF: conception and design of the manuscript, analysis, critical revision, and final approval of the version.

### Conflict of interest statement

The authors declare that the research was conducted in the absence of any commercial or financial relationships that could be construed as a potential conflict of interest.
